# Caring for resettled refugee children in the United States: guidelines, challenges and public health perspectives

**DOI:** 10.3389/fpubh.2023.1046319

**Published:** 2023-09-25

**Authors:** Binh Phung

**Affiliations:** ^1^Department of Pediatrics, Oklahoma State University Center for Health Sciences, Tulsa, OK, United States; ^2^Department of Epidemiology and Public Health, Yale University, New Haven, CT, United States

**Keywords:** resettled refugee children, refugee children, forcibly displaced children, refugee health screening, public health refugee

## Abstract

The global refugee crisis has become an urgent, pressing humanitarian issue, with an estimated 37 million children forcibly displaced from their homes due to conflict, persecution, violence and other human rights violations by mid-2022. Of these children, only a small percentage are eligible for resettlement in a new country. This narrative review examines the physical health needs of resettled refugee children (RRC) in the United States. By analyzing nutrition and growth, infectious diseases, and general health care/screening measures, a set of comprehensive, evidence-based guidelines and public health perspectives are formulated to facilitate ongoing discussion to ensure that RRC receive equitable health care access. An urgent call to action emphasizes cross-border collaboration between governments, public health experts, refugee populations, and disease preparedness authorities in order to prioritize the physical health of RRC. This review will provide primary care providers, public health professionals, social service workers, and community advocates with up-to-date recommendations to meet the health needs of RRC in the U.S.

## Introduction

The global refugee crisis has reached unprecedented levels, with the United Nations High Commissioner for Refugees (UNHCR) estimating that *37 million children*^1^ have been *forcibly displaced*^2^ from their homes by mid-2022 due to political conflict, persecution, violence, and other human rights violations (1, 2). Of those who had resettled in a different country, one-third are comprised of children under the age of 18, while adults accounted only a fifth of displaced individuals (2). Despite this large disproportionate representation, only a small percentage of the forcibly displaced children (less than 4%) are eligible for resettlement in a new country (1). Thus, it is imperative to focus on the physical health needs of resettled refugee children (RRC) in order to ensure that they have equitable access to resources and support.

When discussing the refugee crisis, it is essential to distinguish amongst *refugees*, *migrants*, and *immigrants*, as each holds different legal statuses. Refugees are fleeing their homelands due to conflict, persecution, or violation of human rights, and are entitled to international protection. Most refugees are considered “*externally displaced*,” meaning they have been forced to abandon their home country but may not necessarily have asylum in a new country. Migrants, conversely, are choosing to move for reasons such as career, education, or family reunification, and may also be fleeing poverty, political instability, or gang violence.

This narrative review focuses on three physical health domains including nutrition and growth, infectious diseases, and general health care/screening measures of RRC living in the United States. By analyzing these domains, a summary of evidence-based guidelines and public health perspectives are formulated to ensure that RRC receive equitable health care access and support. Among the 15 countries hosting the majority of the world refugee children, the U.S. is home to an estimated 3.3 million of them (2). Thus, U.S. primary care providers, public health professionals, social service workers, and community advocates should be knowledgeable about how to properly assess and provide care for refugee children upon their arrival.

## Methods

### Search strategy

The narrative review followed systematic search methods to identify original studies, meta-analytic reviews, longitudinal assessments, and population health profiles of refugee children who had resettled in the United States from 2010 to 2021. A search of the PubMed, EMBASE, MEDLINE, and Web of Science databases was conducted on March 28, 2022, with an updated search performed on February 22, 2023. Keywords used in the searches included “*resettled refugee children,”* “*refugee children,”* “*health considerations for refugee children,”* “*initial domestic medical examination,” “refugee health screening,” and “public health implications.”*

### Eligibility criteria

Initially, 3,750 titles/abstracts were identified, which were then uploaded to the Covidence database, an online systematic review software. Here, two independent reviewers used an automated selection instrument to review the keywords, titles, abstracts, and full-text reports. Automated tools removed the majority of records due to lack of context for the United States.

**Figure 1:**
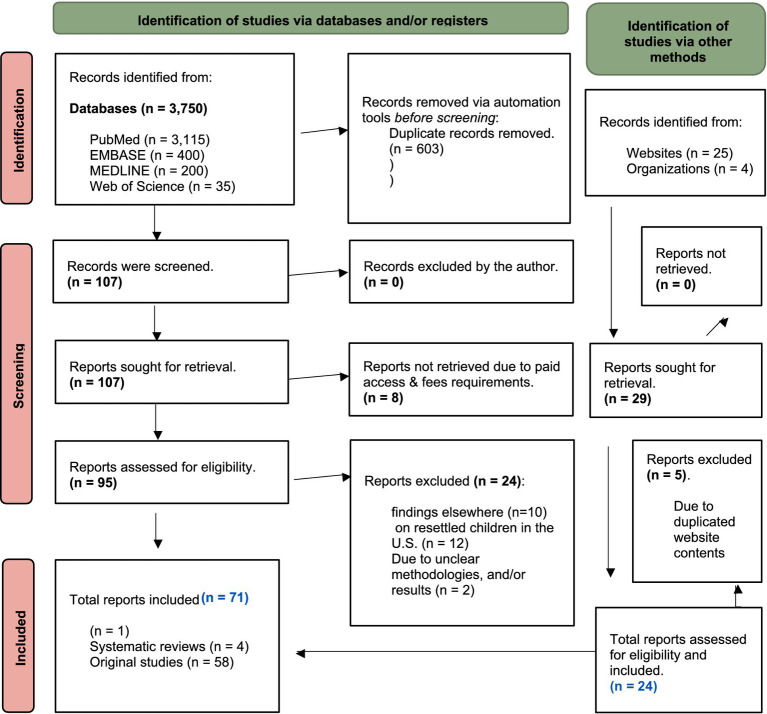
PRISMA flow diagram for new systematic reviews searches.

Through the screening process, the selection was narrowed to 105 records. Subsequently, 71 records including 1 meta-analysis, 4 systematic reviews, and 58 original studies were identified as meeting the eligibility criteria (Table 1). Further evidence collection involved manual searches of websites and organizations, yielding 26 additional reports for the final analysis (e.g., refugee health profiles, global trend statistics, overseas health screenings/assessments, pre-departure vaccination programs, guidelines/recommendations, and policy-specific topics). This review was conducted in accordance with the 2020 PRISMA statement (3) for reporting new systematic reviews, as portrayed in the PRISMA Flow Diagram for New Systematic Reviews Searches (Figure 1).

**Table 1:** Eligibility criteria.

Inclusion criteria	Exclusion criteria
Child refugee data & statistics [birth – 18 years], direct observations, and insights from overseas expert panel clinicians	Publications that did not clearly define age [birth – 18 years old]
Physical health, health examinations, guidelines/recommendations for RRC after their resettlement in the U.S.	Mental health, psychosocial issues, developmental health, and cognitive health of RRC
Multi-state (or multi-regional reports) that provided longitudinal assessments, and/or health profiles of RRC *after* their resettlement in the U.S.	Screening and/or guidelines for international adopted children
Published in English language	Non-English language
Free article access	Articles with restricted and/or paid access

## Results

Thematic synthesis of meta-analyzes, systematic review findings, up-to-date guidelines, and qualitative research data were summarized pertaining to the three physical health domains associated with caring for RRC in the U.S.

### Nutrition and growth

In the Appendix section (Supplementary Table S1), anthropometric definitions, measurements, and classifications of nutritional statuses (e.g., wasting, stunting, overweight, and obesity) are included.

#### Undernutrition v. overnutrition

To assess the prevalence of *undernutrition* (i.e., wasting and stunting) and *overnutrition* (i.e., overweight and obesity) among RRC from birth to 10 years of age, a 2012–2014 U.S. study utilized anthropometric measurements such as BMI, weight, length and/or height from *overseas medical examination* (OME) of newly RRC, and compared the data to that of low-income children of the same age in Washington state (4). As per World Health Organization (WHO)^3^ standards for *children 0–2 years old* and Centers for Disease Control and Prevention (CDC)^4^ standards for *children > 2 years old* (5, 6), about 45% of these RRC met criteria for at least one form of malnutrition (ranging from *wasting* to *stunting*) (4). When grouping the RRC by their country of origin, Somali and Iraqi children ages 5 to 10 years had the highest prevalence of wasting (low ‘weight-for-height’), while Burmese children of the same age had the highest prevalence of stunting (low ‘height-for-age’) (4).

Refugee children arriving from certain areas of the world are also at risk of overnutrition (7). Pre-departure data of RRC from Syria in 2015–2016 showed that 11% of these children aged 6–59 months were overweight or obese (8). Iraqi children who had resettled in Washington state were found to be more likely to be obese than their conationals (children of the same country of origin) who were 5 years old or younger (4). By comparison, there was no statistically significant difference in obesity between younger and older children from Somalia and Burma. There are numerous factors which likely contributed to weight gain amongst RRC in high income countries (HICs), such as the adoption of a Western diet (7), decreased physical activity, parental perceptions of food safety (or lack thereof), cultural beliefs and values (9), level of acculturation to U.S. lifestyles (10), poor quality of housing (11), and increased food availability (12). Despite these general trends, differences observed across different countries of origin suggest that the nutritional needs of RCC vary.

#### Micronutrient deficiencies

Studies on RRC have shown that they may be at risk for developing deficiencies in certain micronutrients such as iron, vitamins A, B12, and D. In a systematic review conducted by Baauw et al. (13), it was discovered that RRC in HICs (e.g., United States, United Kingdom, Australia, Canada, and Germany) were at an increased risk for these micronutrient deficiencies. In 2011, a study specifically identified deficient absorption of vitamin B12 among Bhutanese refugee children resettled in three separate states (Texas, Utah, and Minnesota). This could be due to the lack of dietary options such as meat, eggs and dairy products, which were more commonplace their country of origin (Nepal) (14). Vitamin B12 deficiency is especially important as it can lead to neurological regression and megaloblastic anemia if left untreated (15). Supplementary Table S3 gives a list of common micronutrient deficiencies, associated signs/symptoms, and recommended labs.

### Infectious diseases

Refugee children who originate from low-income countries (LICs) have an increased risk of developing infectious diseases (IDs) due to endemic diseases within their native countries, barriers in accessing healthcare, unaffordability of public health services, as well as lack of vaccinations and inadequate nutrition. Additionally, violence and trauma can further exacerbate these issues. To ensure that these RRC receive a timely diagnosis and appropriate treatment for IDs, U.S. healthcare professionals should be informed of the prevalent IDs among refugee groups. This section highlights some of the more common IDs, although the list is not exhaustive. Supplementary Table S4 provides detailed information on pre-departure screening, post-arrival assessment requirements, and recommended post-arrival lab testing for tuberculosis, hepatitis B virus, intestinal and tissue invasive parasitic infections.

*Tuberculosis* (TB) – Tuberculosis (TB) has been designated as a *Class A*^5^ disease, with refugees not being allowed to enter the U.S. until properly treated (16). Studies have found that the *country of birth* is a significant risk factor for new active TB infections (17). In the U.S., *latent TB infection* (LTBI) is much more common than active TB, with 80% of TB cases stemming from longstanding, untreated latent infections (18). A 2010 report found that 12% of RRC arriving in the U.S. were diagnosed with LTBI during their OME (19). Therefore, the CDC recommends that RRC aged 2–14 from *endemic* or *high-burden disease* countries (i.e., countries with TB incidence rate of **≥**20 cases per 100,000 population) should be screened with an *interferon-gamma release assay* (IGRA) blood test (20), while adolescents over 15 years old should be screened with chest radiographs (17). Some unique issues associated with IGRA^6^ are discussed in the footnote.

*Hepatitis B virus* (HBV) – Hepatitis B virus (HBV) infection is preventable in the U.S. through childhood vaccination programs yet still poses a public health problem for RRC. Exposed children are more likely than adults to become chronically infected with HBV, which may lead to severe liver disease (22). A comprehensive literature review on the international spread of HBV showed that there was a lack of robust epidemiological and surveillance data in LICs (23). Mitruka et al. (23) conducted a study in the U.S. that suggested certain at-risk communities were not routinely being screened for HBV in four states (California, Massachusetts, Minnesota, and Washington). Fortunately, it appears that HBV vaccine coverage has generally improved the prevalence of HBV infections among US-bound refugees. Yun et al. (22) reported improvements in the prevalence of HBV among RRC living in Minnesota, Pennsylvania, and Washington state from 2006 to 2012 and attributed these positive findings to higher rates of HBV vaccination in these states. The CDC recommends vigilance in screening all RRC under 18 years old born in (or have lived in) countries with *intermediate* (2 to 7%) or *high* (≥ 8%) prevalence of chronic HBV infection, should they not have a documented negative blood test (24).

#### Intestinal and tissue invasive parasitic infections

Intestinal parasitic infections, such as *Ascaris lumbricoides*, *Trichuris trichiura*, and hookworm species, and *Strongyloides stercoralis*, are widely reported among refugees and are typically associated with eosinophilia (25). According to the cross-sectional study conducted by Webster et al. (26) on 1,335 refugees coming to the U.S. from Thailand, the prevalence of these infections was strongly linked to *age* (beginning in infancy to 2 years of age, and peaking in adolescents 12–17 years of age). Even though infections caused by nematodes (e.g., *Ascaris lumbricoides*, *Trichuris trichiura*, hookworm species) as well as *Strongyloides stercoralis*, are commonly associated with eosinophilia, this study suggested that eosinophilia was not always a strong predictor of infection (26).

A retrospective cohort study (2012) involving 26,956 refugees from Africa and Southeast Asia who resettled in Minnesota showed that a single-dose of albendazole (antiparasitic), given overseas as *presumptive pre-departure therapy* (PPDT),^7^ significantly decreased the prevalence of parasitic infections (e.g., helminths, schistosomiasis, and malaria) (27, 28). It was found, however, that certain infections, such as *Giardia intestinalis* (giardiasis) and specific strains of *Plasmodium ovale*/*Plasmodium vivax* (malaria), were not susceptible to this standard single-dose albendazole PPDT (27). Interestingly, malaria outbreaks in endemic parts of Southeast Asia were often clustered along international borders with complex ecological interactions between the landscape, humans, mosquito vectors, and particular *Plasmodium* species. This created additional challenges for local health officials administering PPDT for refugees departing from these border regions (e.g., Thailand-Myanmar border) (29).

#### Vaccine-preventable diseases

Vaccine-preventable diseases (VPDs), such as measles, polio, meningococcal meningitis, yellow fever, hepatitis A, and cholera, have been a major health concern in refugee resettlement processes in the past (30, 31). In 2009, a child died and an infant was born with congenital rubella syndrome due to successive outbreaks of measles, rubella, and varicella among US-bound Liberian refugees from *Cote d’Ivoire*, and travel suspension of refugees from those transit camps was enforced for 6 months (32). In light of the resurgence of more than 18,000 measles cases in 2022, leading to 142 deaths of newly resettled Afghan children over the course of 3 months, the World Health Organization (WHO) reported heightened safety concerns for resettling communities across the globe (33). The infection rate of COVID-19 among RRC in the U.S. is not available for analysis, but is being monitored on the Migration Data Portal which includes surveillance data for refugees and migrants from over 20 countries with the highest number of COVID-19 cases.

The U.S. Refugee Admissions Program (USRAP) Vaccination Program, launched in 2012 in partnership with the International Organization for Migration (IOM), has been a breakthrough in limiting overseas VPD outbreaks (e.g., tuberculosis and measles) in the places hosting US-bound refugees (30). The USRAP Vaccination Program is offered voluntarily to refugees at the time of their OME in more than 80 participating countries (30). The program’s vaccination schedule currently consists of 11 vaccines that protect against 14 VPDs (e.g., measles, mumps, rubella, diphtheria, tetanus, pertussis, hepatitis B, polio, varicella, and others). During the COVID-19 pandemic, when some global immunization services for children were reduced and/or suspended (34), including here in the U.S., the USRAP Vaccination Program was still serving a critical role in safeguarding migrating refugee populations (30).

### General health care/screening measures

#### Lead screening

In October 2021, the Centers for Disease Control and Prevention (CDC) revised the standard acceptable *blood lead reference value* (BLRV) from 5.0 μg/dL to 3.5 μg/dL (35), leading to more individuals being identified with potential “lead exposure.” Data from domestic refugee children collected under the previous BLRV of 5.0 μg/dL revealed higher concentrations of *blood lead levels*^8^ (BLLs) than US-born children (36, 37). Of the 27,000 refugee children aged 6 months to 16 years residing in 11 states (Colorado, Idaho, Illinois, Indiana, Kentucky, Massachusetts, Minnesota, North Carolina, New York, Texas, Utah, and Washington), a cross-sectional study demonstrated 19% had BLLs >5.0 μg/dL (36). Further analysis highlighted that males RRC from India, Afghanistan, Burma, and Nepal had an increased prevalence of BLLs ≥5 μg/dL (36). Seifu et al. (37) reported that *microcytosis*, *male sex*, and *young age* were the strongest predictors of having elevated BLLs ≥5 μg/dL among newly arrived refugee children. The CDC advises initial screening *via* a blood lead level test for all those under 16 years of age, plus all pregnant/lactating people within 90 days of their arrival in the U.S. Follow-up testing is recommended (i.e., a blood lead level should be repeated for all RRC ≤ 6 years old) around 3–6 months after resettlement regardless of the initial screening result (35).

#### Anemia

Anemia is another common diagnosis among refugee communities in the U.S. (38, 39). This condition is usually the outcome of inadequate nutrition and iron deficiency, but other causes may include vitamin B12 deficiencies (40), undiagnosed metabolic disorders (6), chronic gastrointestinal infections (hookworms), and hematologic disorders [βeta-thalassemia (41)].^9^ Refugees from LICs, where anemia and other blood dyscrasias are prevalent, could experience additional health disparities in the host country (or country of first asylum) due to lack of resources, capacity and/or policy restrictions. A list of common micronutrient deficiencies, their associated signs and symptoms, and recommended lab tests can be found in Supplementary Table S3.

#### Dental health

Refugees and immigrants coming to the U.S. are particularly vulnerable to developing dental caries (42). A 2020 systematic review identified affordability, awareness and access as the three main barriers to dental care for refugees and their families living in HICs (e.g., the United Kingdom, United States, Canada, and Australia) (43).

## Discussion

### Perspectives on nutrition and growth

#### Issues: global nutritional challenges and growth patterns of refugee children

RRC originating from the Middle East (e.g., Syria, Afghanistan), and North Africa (e.g., Egypt, Libya, Lebanon, Yemen) living in surrounding LICs face a variety of nutritional challenges and suboptimal growth patterns. Undernutrition,^10^ coupled with the COVID-19 pandemic and Russia’s ongoing war in Ukraine (44), further complicates the situation. Their lack of nutrition compromises RRC’s ability to adjust to different dietary needs and access existing nutritional programs. Over time, this may lead to long-term consequences on their physical, developmental, and mental health issues. Although a lot of research is dedicated to undernutrition in RRC under 5 years old, there is a lack of studies exploring nutrition and micronutrient deficiencies among older children and adolescents (5). Thus, it is important that U.S. healthcare providers be aware of the potential nutrition-related risks that RRC may face and take appropriate steps to monitor and address malnutrition (6, 45).

The research conducted on RRC living in HICs has found that their risk for undernutrition and overnutrition (overweight/obesity) is elevated (46–48). For instance, a comparison of RRC aged 0–16 years old from Washington and Pennsylvania and non-refugee, low-income control sample showed a higher prevalence of obesity in the refugee group (46). Similarly, refugees aged 2–18 years from Africa and Southeast Asia in Rhode Island experienced a doubling in overweight/obesity from 17 to 35% after 3 years of resettlement (47). Olson et al.’s 2017 longitudinal study at the New York SUNY medical clinic found that whilst both groups, refugees and non-refugee control, increased in overweight/obesity after 9 years, refugees were disproportionately more likely to develop these health conditions (48). It is unclear whether this perceptible rise in overweight/obesity is a reflection of the global increase in obesity, or caused by a shift in the economic status of newly arriving refugees. Nevertheless, it is clear that refuges are increasingly susceptible to both undernutrition, which may lead to cognitive and physical developmental delays in childhood, and overnutrition (overweight/obesity), a risk factor associated with numerous chronic health conditions in adulthood (49).

#### Remedies: cross border collaboration and food security interventions

In order to alleviate the adverse impacts of displacement, armed conflicts, and natural disasters, it is prudent for governments, organizations, and public health authorities to take proactive steps to provide refugees with secure access to safe and nutritious foods. Lutfy et al. (42) stressed that the public health community prioritize a patient-centered approach along with enhanced nutritional monitoring and screening measures. Additionally, correlating nutritional data with domestic resettling agencies could enable the development of nutritional monitoring interventions tailored to the age, demographics, and growth parameters of different groups of refugee children (45). Cross-border collaboration between stakeholders and host communities is key to achieving improved food security among refugees, and initiatives such as the Global Food Security Cluster (50) can help facilitate this. Evidence-based food security interventions, tailored to the needs of refugees, should be implemented in order to prevent and address under- and overnutrition. The scoping review conducted by Nisbet (51) found that while food security interventions are effective in assisting refugees, they are often constrained by scope and length of program implementation. Other public health programs, media campaigns, and public education measures—such as those launched by the United Nations World Food Program (52) (WFP)—are essential in creating awareness of domestic food insecurity among refugees. If cross-border collaboration is successful in providing evidence-based solutions, raising awareness, and deepening understanding of the domestic food security context, then the nutritional health and well-being of refugees can improve significantly. Therefore, continued and research and investigation into these matters is necessary.

### Perspectives on infectious diseases

#### Issues: the global risk of tuberculosis in refugee populations

##### Tuberculosis

The global TB crisis is one that continues to worsen, with an estimated 10 million new cases being reported in 2020 (53). In the U.S., the proportion of foreign-born TB cases has been steadily increasing for two decades, and now makes up 65–70% of all reported cases according to data compiled in 2018 (54, 55). Young children are vulnerable to TB and are more prone to developing severe TB disease as compared to adults (56). Cases of *multi-drug resistant TB* (MDR-TB) have also been reported in various regions including India, South Africa, Somalia, Kenya, and Syria (57). The rise in MDR-TB in Syria was attributable to high pre-civil war MDR-TB rates, war-damaged healthcare infrastructure, and poor hygiene conditions (58). Latent TB infection (LTBI) is another mounting public health issue due to lack of resources and/or inadequate infrastructure in many low- and middle-income countries. From 2019 to 2020, Bangladesh, Guatemala, India, Zimbabwe, Afghanistan, Ethiopia, South Sudan, and other countries reported inadequate resources and lack of infrastructure as the two most common barriers to proper treatment of LTBI (and active TB) (59–65). To address this modern challenge, we must continue to invest in detection, control, and prevention measures to mitigate the spread of TB worldwide.

#### Remedies: public health strategies to combat the global TB crisis

The magnitude of tuberculosis (TB) as a global risk is now widely recognized and considerable efforts are being taken to curtail it. In the U.S., one of the more cost-effective, high-yield strategies to prevent importation of TB cases and contribute to the elimination of disease is utilizing the overseas medical examination (OME) to provide voluntary testing and to initiate treatment of LTBI, particularly for individuals departing from a high-disease burden country (66). Since 2007, clinicians and staff from 159 countries have provided invaluable support in the implementation of the U.S. Tuberculosis Technical Instructions (TBTI). This includes requirements for chest radiographs in US-bound refugees aged 15 years and above, prior to entrance to the U.S., as well as sputum smear and culture, drug susceptibility testing for those with abnormal radiograph findings, and tests for HIV plus other concurrent symptoms that might indicate TB (30). Evidence suggest that investment in TB control program in source countries with a high-disease burden has been more beneficial than enhancing TB screening algorithms alone (67). Demonstrably, the implementation of TBTI has fostered the growth of laboratories which have the capacity to perform TB cultures and tests for second-line TB drug resistance and multidrug-resistant TB (MDR-TB) (68).

The global collective public health strategies – The Tuberculosis Action Plan (69) (WHO European Region 2016–2020), Wolfheze Consensus Statement (69), and the European Respiratory Society–WHO TB Consilium (70, 71) – have been effective in mitigating the spread of TB. The Tuberculosis Action Plan seeks to strengthen coordination between European countries to reduce the burden of TB. The Wolfheze Consensus Statement (2016) advocates for improved access to diagnosis/treatment, better surveillance systems, improved care delivery, and international collaboration. Similarly, the European Respiratory Society-WHO TB Consilium (2017) focuses on prevention and control of TB. To ensure further progress is made on this issue, more public health research is needed to evaluate the impact of these strategies in countries with high TB prevalence. Such research should also seek to build collaborative partnerships between high- and low-income countries.

#### Issues: the global risk of hepatitis B virus in refugee populations

##### Hepatitis B virus

Refugees and those forcibly displaced bear an especially high burden of HBV-related infections. In children, the risk of disease progression from acute to chronic HBV infection is *inversely* linked to the *age* at the time of infection (72). For example, greater than 90% of infected infants (birth to 1 year), 25–50% of infected children (aged 1–5 years), and less than 5% of infected older children and adults can progress to chronic HBV infection (which can lead to cirrhosis, liver failure, and hepatocellular carcinoma) (73). HBV transmission during the perinatal and early childhood period is a major contributor to the global HBV burden in *intermediate* and *high* prevalence countries (73). As a result, ensuring a high screening rate for HBV infections in countries receiving refugees could have a transformative public health impact. Taking into account the general underdiagnosis of HBV infection by medical professionals in the U.S. (73), the initial domestic medical examination (DME) is an opportune time to identify/diagnose and prevent HBV infection.

#### Remedies: unified global public health responses to VPDs

The increasing emergence of vaccine-preventable diseases (VPDs) among refugee populations requires a unified public health response at the global level that takes into consideration the current migratory patterns and shifts in the geopolitical landscape. Such a response necessitates a comprehensive assessment of both acute and chronic VPDs, coupled with the implementation of robust surveillance programs and awareness campaigns to promote proactive vaccination strategies. By investing in domestic and international public health partnerships, potential costly reactive measures that are often taken in response to VPD outbreaks can be minimized. An example of this is the 16 outbreaks and 107 confirmed cases of imported measles in California in 2011, which were estimated to have cost local and state public health departments between $2.7 to $5.3 million USD (74).

In-depth epidemiological research is urgently needed to inform a better understanding of VPDs and related vaccination and treatment adherence amongst diverse, conflict-affected, forcibly displaced populations (22). RRC can be especially vulnerable to the risks posed by VPDs, given their exposure to – and potential intensification of – environmental factors such as mass migration, overcrowding, and lack of clean water/sanitation facilities (31). Refugee populations often have limited access to vaccinations and medical services in both their home and host asylum countries. To ensure a successful unified public health response to VPDs, there must be a cohesive effort to improve the overall health and well-being of forcibly displaced children, supported by public health initiatives and enabled by ongoing collaborations and contributions of reliable epidemiological data between public health planners, refugee populations, and organizations focused on disease emergence preparedness (75).

### Perspectives on general health care & medical screening

#### Issues & remedies: effectively promoting health and well-being of refugees

The Division of Global Migration and Quarantine^11^ (DGMQ), part of the Centers for Disease Control and Prevention (CDC), provides instructions for the *overseas medical examination* (OME) of refugees prior to their departure to the U.S. (75). The primary purpose of the OME is twofold – to ensure that refugees entering the U.S. pose no public health threats, and to identify any existing health conditions requiring ongoing medical attention post-resettlement (76). To assist state public health partners, the CDC has also created a set of guidelines (not mandates) to assist healthcare professionals perform the initial *domestic medical examination* (DME) within 90 days of entry in the U.S. (75). Supplementary Table S4 details the pre-departure screening process, post-arrival assessment requirements, and suggested disease-specific lab testing provided by the DGMQ/CDC.

These guidelines developed by the DGMQ/CDC comprise evidence-based clinical recommendations and checklists to support receiving states with the initial DME. Aspects covered include the history and physical examination, screening for hepatitis and HIV infection, domestic immunization guidelines, guidance for evaluating nutritional status, and testing for blood lead levels, sexually transmitted diseases, tuberculosis, malaria, and intestinal parasites (75). These recommendations for the DME are not meant for ongoing continuity of care, but rather to emphasize the health conditions that need be immediately assessed and taken care of during the first 90 days of entry. For the purposes of recognizing prevalent health conditions among US-bound refugees, the DGMQ/CDC has created “Refugee Health Profiles.” These profiles include pertinent details and information on different refugee groups such as population background, demographics, health conditions, routes of movement and asylum, healthcare status prior to entry, and post-arrival medical screening (77).

The public health program in Denmark serves as a successful example of the impact of providing early General Health Assessments (GHAs) to refugees living in a HIC. This program provides free GHAs to all newly arrived refugees in Aarhus and Copenhagen (76, 78). Medical and public health professionals agree that early GHAs (similar to the DMEs in the U.S.) are beneficial for promoting the health of refugees and their integration into their new commumities (78). In light of this, it is important for U.S. state/local health departments and medical clinics to consider fundamental requirements and best clinical practices when carrying out the initial DME. These include implementing a patient-centered approach, reducing health disparities and cultural barriers, improving access to primary healthcare services, explaining consent and confidentiality, and providing support for refugee advocacy/community resources to promote health and well-being (79).

### Limitations

#### Selection bias

This narrative review was limited to English language publications, likely leading to a bias towards clinical guidelines and epidemiological descriptive analyzes from English-speaking HICs. The data extraction, categorization and data quality assessment were completed solely by the author, further contributing to selection biases in the narrative commentaries. While this review concentrated on three physical health domains, the ongoing consequences of COVID-19 on food insecurity, poverty (80), and other social determinants of health were not covered despite their potential impact on the overall health of refugee children. Acculturation was overlooked as it is difficult to define and measure within the scope of this narrative.

#### Omission of mental and developmental health

Mental and developmental health are critical issues, particularly for refugee children, and yet this review omitted titles and abstracts containing relevant words and phrases such as ‘mental health’, ‘psychosocial issues’, “developmental health,” “cognitive health,” which may have resulted in a shallow representation of the mental and psychological traumas experienced by these children. Throughout the process of forced migration (*pre-, peri-, post-*), refugee children are subjected to a variety of physical, mental, and psychosocial stressors, and are often accompanied by post-traumatic stress disorder (PTSD), anxiety, depression, emotional, and behavioral issues (81–83). Research by Fazel et al. (81) highlighted the influence of violence exposure on the mental health and developmental capacity of RRC depending on their migration period. Yet, these points are not comprehensive enough to draw broad conclusions about all RRC living in the U.S. More thorough examination of mental health literature and translation of qualitative data into descriptive themes should be conducted to provide a nuanced understanding of the mental health and developmental outcomes of RRC living in the U.S.

This review also did not evaluate the adverse risks, harms, and emotional burdens that refugee children could have encountered during their hazardous migration. Reports from the Yale Humanitarian Research Lab (HRL) have revealed that Russia is operating a large-scale, organized network of at least 43 camps and facilities, which has since the onset of Russia’s War in Ukraine in February 2022, detained around 6,000 children in Russia-occupied Crimea and mainland (84). Prolonged exposure to such adverse childhood experiences is likely to inflict long-term health effects on the mental and developmental capabilities of children. The author did not find any clear baseline estimation of the prevalence of PTSD and depression among RRC living in the U.S. for comparative analysis, which was supported by Montgomery (83), who suggested that traumatic-related responses varied significantly depending on prior and/or current exposure to adverse childhood events. Thus, it is prudent to ascertain the prevalence of PTSD and depression among RRC and delve further into the consequences of adverse childhood experiences such as organized camps and facilities in order to gain insight and provide appropriate evidence-based practice recommendations.

## Conclusion

The global refugee crisis disproportionately affects children, yet only 4% of these refugee children have resettlement opportunities. Nutrition is an area of significant concern, with both under- and overnutrition becoming common issues in the U.S. Likewise, the additional threat of micronutrient deficiencies adds to the importance of providing tailored public health initiatives and food security intervention programs to different age groups, population demographics, and growth-specific parameters. The increased risk of illness due to tuberculosis, hepatitis B virus, and vaccine-preventable diseases also requires more research into the epidemiological data of refugee populations to inform effective, systematic interventions. To facilitate this, cross-border collaboration between local/global governments, public health experts, refugee populations, and various organizations focused on disease emergence preparedness must occur. Finally, healthcare professionals are vital to managing refugee health both domestically and abroad. The role of primary care providers, public health officials, social service workers, and community advocates is indispensable in bridging refugee children with the U.S. healthcare system.

## Author contributions

BP: conceptualization of mini systematic review, methodology, formal analysis, writing – original draft preparation, and writing – review and editing.

## Footnotes

^1^This number includes some 12.5 million child refugees [i.e., 10.8 million under ‘UNHCR mandate’ and children from Venezuela displaced broad, and 1.8 million Palestine children registered with UNRWA, 1.2 million asylum-seeking children], and 22.8 million children internally displaced within their own countries (UNRWA is United Nations Relief and Works Agency for Palestine refugees registered in Jordan, Lebanon, Syria, Gaza, and the West Bank). More information available at: https://www.unrefugees.org.uk/wp-content/uploads/Global-Trends-2021.pdf.

^2^The terms forcibly displaced, externally displaced, refugees, migrants, asylum seekers describe individuals who: i) are on the move, ii) have left their countries, and iii) have crossed country borders.

^3^Anthropometric definitions based on the World Health Organization (WHO) multicenter growth reference study for children ages 0 to 2 (5).

^4^Anthropometric definitions based on Centers for Disease Control and Prevention (CDC) guidelines for children over age 2 (6).

^5^Class A include diseases and health conditions that render a refugee *ineligible* to enter the U.S. until that individual has been treated. Class A includes active TB, syphilis, gonorrhea, Hansen’s disease (leprosy), and mental disorders *with* harmful behaviors (15).

^6^In the U.S. (and other high-income countries), there are some issues pertaining to the use of *interferon-gamma release assay* (IGRA): 1) performance and reporting of results in a lab setting fall under the auspices of regulatory certification, 2) inconsistent reproducibility of test results (23), and 3) lack of consensus on “normal” threshold values (19).

^7^Presumptive pre-departure therapy for intestinal parasites includes: albendazole, ivermectin, praziquantel (27,28).

^8^Blood lead levels (BLLs) ≤10 μg/dL ➔ adverse cognitive and behavioral development (35). Above 40 μg/dL ➔ headaches, abdominal pain, anorexia, constipation, clumsiness, agitation, lethargy. At 70 μg/dL ➔ seizures, ataxia, mental status changes, coma, death (35).

^9^βeta-thalassemia (41) which is thought to carry a prevalence of 1 to 1.5 million Afghanistan, could have ongoing public health considerations given the unprecedented evacuation of Afghanistan refugees in August 2021.

^10^There are 4 types of undernutrition: 1) acute malnutrition, AKA “wasting” (i.e., low *weight-for-height*); 2) chronic malnutrition, AKA “stunting” (i.e., low *height-for-age*); 3) micronutrient deficiencies, and 4) underweight (i.e., low weight-for-age, a child who is underweight may be stunted, wasted, or both) (3).

^11^These instructions are developed in accordance with Section 212(a)(1)(A) of the Immigration and Nationality Act (INA) (16).
