# Supplementary material for: Caring for resettled refugee children in the United States: guidelines, challenges and public health perspectives
Source: Front Public Health. 2023 Sep 25;11:1046319. doi: 10.3389/fpubh.2023.1046319 (PMC10561301; doi:10.3389/fpubh.2023.1046319)
Supplement: Supplementary file 1 [file Data_Sheet_1.docx]

**APPENDIX**

**Anthropometric Definitions of Nutritional Status**

The following are specific definitions of nutritional status (5, 6) used in this review:

- Wasting: Acute malnutrition, also known as wasting, is based on a comparison of the child’s *weight-for-height* with the number of standard deviations (z-scores) from the median for a child of the same age and gender in a reference population
- Stunting: Chronic malnutrition, also known as stunting, is assessed by comparing the child’s *height-for-age* with the number of standard deviations (z-scores) from the median for a child of the same age and gender in a reference population. Chronic malnutrition is defined as height-for-age z-scores < -2.0.
- Overweight and obesity: overweight and obesity status is assessed by calculating a ratio of the child’s *weight-for-height* and comparing the ratio to the number of standard deviations (z-scores) from the median for a child of the same age and gender in a reference population.
- For children below 2 years of old, their growth indicators should be compared to World Health Organization (WHO) standardized growth references, while those over 2 years old should use the Center for Disease Control and Prevention/National Center for Health Statistics (CDC/NCHS) references (6).

**Table S1 –** Nutritional Status Categories: Anthropometric Definitions (5, 6)

| Nutritional Status Category | Age 0 to 1.99 years | | | Age 2 to 10 years |
| --- | --- | --- | --- | --- |
| Chronic malnutrition | | Length-for-age < 2.3rd percentile | Height-for-age < 5th percentile | |
| Acute undernutrition | | Weight-for-length < 2.3rd percentile | BMI < 5th percentile | |
| Healthy weight | | Weight-for-length > 2.3rd percentile and < 97.7th percentile | BMI > 5th percentile and < 85th percentile | |
| Overweight | | n/a | BMI > 85th percentile and < 95th percentile | |
| Obesity | | Weight-for-length > 97.7th percentile | BMI > 95th percentile | |

**Class A and B health conditions** (85)

Applicants with Class A conditions are *inadmissible* such as infectious tuberculosis (Class A TB), infectious syphilis, gonorrhea, and infectious Hansen’s disease. Applicants with Class B conditions are still *admissible* but might require treatment or follow-up for tuberculosis. Class B TB includes those who completed successful treatment overseas for TB disease (Class B0), those with signs suggestive of TB but whose overseas lab tests ruled out current infectious TB disease (Class B1), those with a diagnosis of LTBI (Class B2), and the close contacts of persons known to have TB disease (Class B3). After arriving in the U.S., a follow-up TB examination is recommended for persons with Class B1 and B2 TB (85).

**Table S2** – The Resettlement Process: Completion of Overseas Medical Examination (28) (OME) and Domestic Medical Examination (DME) (86)

| Overseas Medical Examination (OME): History & Physical Exam | | |
| --- | --- | --- |
| - All immigrants, refugees, and others applying for permanent residence in the U.S. must undergo an *overseas medical exam* (OME) before being accepted for resettlement (28). - The OME is valid 6 months before departure and is conducted according to technical instructions from the CDC’s Division of Global Migration and Quarantine (DGMQ) (28). - The results of the medical examination and vaccination history are recorded on official Department of State forms by more than 760 panel physicians appointed by the U.S. consul of the International Organization of Migration (IOM) (28). - The OME is used to identify individuals with Class A health conditions that prevent entry into the U.S. and Class B health conditions that require follow-up soon after arrival (28). - For refugees with tuberculosis or other medical conditions of public health significance, the records are transmitted to DGMQ/CDC’s Electronic Disease Notification (EDN) system (85). | | |
| OME Components (28)   - Medical history - Physical examination - Mental health - Blood test screening for syphilis, and physical examination of other sexually transmitted diseases if >15 years old (positive results may require proper treatment prior to departure to U.S.) - Tuberculosis screening - Review of vaccination records - Other physical or mental abnormality, disease, or disability | **Class A health conditions** (28)   - Class A is a physical or mental health condition that renders a person ineligible for admission or adjustment of status. - Active or infectious tuberculosis (TB) - Sexually transmitted diseases (e.g., untreated syphilis, chancroid, gonorrhea, granuloma inguinale, lymphogranuloma venereum) - Hansen’s leprosy disease (lab-confirmed disease requires 6 months of treatment prior to departure to U.S. and treatment must be continued after arrival) | **Class B health conditions** (28)   - Class B refers to significant health problems affecting ability to care for oneself or attend school/work or that require extensive treatment or possible institutionalization. - Inactive, latent, or non-infectious tuberculosis (TB). - Treated syphilis. - Other sexually transmitted diseases - Pregnancy - Treated Hansen’s leprosy disease. - Sustained, full remission of substance abuse and/or any physical/mental health disorder without harmful behavior or with a history of such behavior unlikely to occur. |

| Domestic Medical Examination (DME): History & Physical Exam |
| --- |
| - Domestic medical examination (DME) is strongly recommended by the CDC to be completed within the first 30 to 90 days after arrival (but not mandated) (86). - Purpose: Eliminate health-related barriers to successful resettlement and ensure follow-up of Class A or Class B health conditions identified during the Overseas Medical Examination (OME). - Completed in the state of initial arrival in the U.S. (86) - DME is coordinated by state-appointed refugee health officials or programs. - Services can be delivered by local health departments, community health clinics, or private healthcare providers.^85^ - History and Physical Examination (H&P) guidelines and templates available at: <https://www.cdc.gov/immigrantrefugeehealth/guidelines/domestic/guidelines-history-physical.html> - General and optional testing for newly arrived refugees (87)   - Complete blood count with a white blood cell differential and platelets  - Urine analysis (if old enough to provide a clean-catch urine specimen)  - Infant metabolic screening in newborn infants (based on individual state guidelines)   - Specific populations (87) - serum lipid profile - cancer screening - uric acid (for Hmong refugees) - Prescription of age-appropriate multivitamin with iron (for refugee children ages 6 and 59 months); empiric treatment with antiparasitic(s). - Referral for specialist consultation, mental health services, WIC (Women, Infants, and Children), and social work services as needed. - In the U.S., healthcare providers who conduct the initial DME are encouraged to adhere to the National Center on Immigrant Integration Policy’s recommendations, which include providing culturally appropriate nutrition counseling, connecting children with a primary healthcare provider who can monitor their growth, development, and nutritional status over time, referring pregnant/birthing people and children under five to the Women, Infants, and Children (WIC) program, and providing nutritional support through resettlement agencies and/or local community partnerships (if available) (6). - Other online resources for clinical assessment for refugees:   CareRef online tool for clinicians conducting a routine post-arrival medical screening.  <https://careref.web.health.state.mn.us/>  CDC’s Domestic Refugee Screening Guidance.  <https://www.cdc.gov/immigrantrefugeehealth/guidelines/domestic-guidelines.html>  Refugee Health Profiles.  <https://www.cdc.gov/immigrantrefugeehealth/profiles/index.html>  American Academy of Pediatrics (AAP) Immigrant Child Health Toolkit (88).  <https://www.aap.org/en/patient-care/immigrant-child-health/>  Supplemental clinical guidance and resources for clinicians and public health professionals working with Ukrainians.  <https://www.health.state.mn.us/communities/rih/about/ukrainianmn.html>  Supplemental clinical guidance and resources for clinicians and public health professionals working with Afghanistan refugees.  <https://www.health.state.mn.us/communities/rih/about/afghan.html> |

**Table S3** – Common Micronutrient Deficiencies, Findings, Symptoms Among Refugee Children (66)

| **Deficiencies** | **Facts and**  **Physical findings** | **Symptoms** | **Additional Information** |
| --- | --- | --- | --- |
| Iron deficiency (89) | - Most common nutritional deficiency globally. Estimated to affect 50% of children in the developing world (particularly prevalent among refugee children) - Results from inadequate bioavailable dietary iron - Groups at high risk:  1. Age 6–24 mo. (period of rapid growth) 2. Breastfed infants > 6 mo. (who are not receiving iron supplementation) 3. Those with a history of infrequent consumption of animal sources of iron  - Nondietary factors associated with deficiency. - Parasitic infections (hookworm, malaria) - Hemoglobinopathies (sickle cell trait/diagnosis, thalassemia) - Chronic infections | - Impaired cognitive functioning and motor development, decreased productivity, fatigue | - Complete blood count with a white blood cell differential and platelets, iron studies - Recommended Daily Iron Intake:  \| **Life Stage** \| **Recommended Amount** \| \| --- \| --- \| \| Birth to 6 months \| 0.27 mg \| \| [Infants](https://ods.od.nih.gov/factsheets/Iron-Consumer/) 7–12 months \| 11 mg \| \| Children 1–3 years \| 7 mg \| \| Children 4–8 years \| 10 mg \| \| Children 9–13 years \| 8 mg \| \| Teen boys 14–18 years \| 11 mg \| \| Teen girls 14–18 years \| 15 mg \| \| Adult men 19–50 years \| 8 mg \| \| Adult women 19–50 years \| 18 mg \| \| Adults 51 years and older \| 8 mg \| \| Pregnant teens \| 27 mg \| \| Pregnant women \| 27 mg \| \| Breastfeeding teens \| 10 mg \| \| Breastfeeding women \| 9 mg \| |
| **Note**: Due to the high-risk nature of their past and current diet, the CDC recommends children aged 6 months to 59 months of age should be prescribed an age-appropriate multivitamin. (6) | | | |
| Vitamin A  Deficiency (90) | - Common in developing countries, seldomly encountered in industrialized or high-income countries (HICs). - Leading cause of preventable blindness among children (500,000 children affected annually). - Established policy in refugee camps for vitamin A supplementation, fortification (but high Vitamin A deficiency rates persist). | - Poor night vision, Bitot spots (areas of abnormal squamous cell proliferation and keratinization of the conjunctiva that can progress to blindness). - Impaired bone growth, dry skin and hair. - Impaired humoral and cell-mediated immunity. |  |
| Vitamin B1  (*thiamine*)  Deficiency (6) | - Plays an important role in energy metabolism and tissue building. - May result from inadequate dietary intake (diets heavy in white or milled rice), altered metabolism (fever, liver disease), or losses (diarrhea) | - May lead to beriberi (anorexia, constipation, fatigue, irritability, memory loss, neuropathy, heart failure) |  |
| Vitamin B12 deficiency (6) | - Bhutanese refuges are particularly at risk. - Results from inadequate dietary intake (found in eggs, meat, milk) as well as impaired absorption and malabsorption. | - Commonly asymptomatic but may exhibit fatigue, weakness, numbness/tingling in the extremities, loss of coordination, ataxic gait | - Laboratory studies reveal macrocytic red blood cells (with or without anemia), hyper segmented neutrophils. |
| Vitamin C  Deficiency (6) | - Most at risk are those with chronic malnutrition and diets devoid of fruits and vegetables. - Outbreaks reported in refugee camps (Somalia, Bhutanese refugees in Nepal, Ethiopia, Kenya, Somalia, and Sudan). | - Results in scurvy and symptoms due to impaired collagen synthesis (e.g., ecchymoses, petechiae, bleeding gums). |  |
| Vitamin D  Deficiency (6) | - Prevalent in both resource-rich and resource-limited countries - Cause is multifactorial (including inadequate dietary intake of calcium and vitamin D, prolonged breastfeeding without supplements) and heavily dependent on nondietary determinants such as:  1. Limited sun exposure (protective/religious clothing, movement to temperate climates, tradition of keeping infants indoors) 2. Increased skin pigmentation | - Rickets |  |
| Vitamin B3/Niacin and tryptophan deficiencies (6) | - Deficiency is known as pellagra, typically occurs in combination with lack of other amino acids and micronutrients. - Results from inadequate dietary intake (most common in areas where corn is the primary constituent of the diet and in areas of South Asia where people eat millet) | - Advanced pellagra (not typically seen in infants and children) may cause a symmetric photosensitive dermatitis (“glove-like rash”), diarrhea, stomatitis, and neurologic symptoms. |  |
| Iodine deficiency (6) | - Affects 31.5% of school age children worldwide. - Most common cause of thyroid disease - Results from inadequate dietary intake - Universal salt iodization has reduced the burden of iodine deficiency. | - Physical and developmental growth abnormalities, goiter. |  |
| Zinc deficiency (91) | - Populations consuming plant-based diets are at greatest risk. - Required for catalytic activity > 100 enzymes | - Growth retardation, loss of appetite, impaired immune function, hair loss, diarrhea, delayed sexual maturation, delayed wound healing. |  |

**Table S4** – Specific Diseases & Lab Testing for Refugee Children during the Domestic Medical Examination (DME)

| **Hepatitis A virus** (24) | - Routine screening for hepatitis A virus (HAV) in asymptomatic children is not recommended. HAV vaccination is recommended for children in accordance with Advisory Committee on Immunization Practices (ACIP) recommendations, as well as select adults. |
| --- | --- |
| **Hepatitis B virus** (24) | - Screen all refugee children and adolescents < 18 years old who were born in (or have lived in) countries with intermediate (2% to 7%) or high endemicity (> 8%) rate of chronic hepatitis B virus [HBV] infection countries (e.g., Africa, Southeast Asia, China, Pacific Islands, Alaska, Peru, Northwest Brazil, Mediterranean Basin, Eastern Europe, Central Asia, Japan, Amazon Basin, Middle East). - Testing should be obtained for **hepatitis B surface antigen** (*HBsAg*), regardless of vaccination history (the only exception is if a negative HBsAg test result is documented on overseas medical forms). - Checking antibody to hepatitis B surface antigen (anti-HBs) and total antibody to hepatitis B core antigen (anti-HBc) may be reasonable in older children coming from countries with high prevalence rates (> 8%) or who are at risk of HBV infection. - All refugee children and adolescents < 18 years of age who were born or have lived in countries where the rate of chronic HBV infection is < 2% should be tested as above, if they belong to one of the following high-risk group (92) - Injection drug users - Persons needing immunosuppressive therapy, including chemotherapy, immunosuppression for rheumatologic or gastroenterological disorders. - Refer to [CDC Website](https://www.cdc.gov/immigrantrefugeehealth/guidelines/domestic/hepatitis-screening-guidelines.html#acute) for complete listing of high-risk groups. - Although screening for HBsAg is recommended for all refugee children from countries with intermediate or high prevalence of HBV infection, some experts do not routinely check anti-HBc or anti-HBs in children from countries with HBV prevalence of < 8% to avoid false-positive results and routine testing in this group is not cost-effective. However, *factors* that increase the pretest probability for past or current HBV infection should prompt testing. These factors include: - Children with an immediate family member, particularly the mother, who is chronically infected with HBV. - Children from a low-prevalence country who are members of a subpopulation with prevalence of > 2% (e.g., some indigenous populations). - Human immunodeficiency virus (HIV)-infected children. - Children who received whole blood products or blood components before migration. - Children who are injection drug users, have multiple sex partners, or have a history of sexual exploitation. - All children < 18 years old who are HBsAg-negative should receive the complete hepatitis B vaccination series according to Advisory Committee on Immunization Practices (ACIP) guidelines. The vaccine series can be started while waiting for HBV screening test results. - Any refugee child who had a potential exposure to HBV within 60 days of the new arrival examination should have repeat testing 3-6 months after arrival.   For more details, resources, overseas pre-departure screening and vaccination, and summary recommendations for post-arrival management of viral hepatitis, visit the CDC:  <https://www.cdc.gov/immigrantrefugeehealth/guidelines/domestic/hepatitis-screening-guidelines.html#acute> |
| **Hepatitis C** (24) | - CDC does not recommend hepatitis C virus (HCV) screening for refugee children < 18 years old during the DME unless they are members of high-risk groups or risk factors, which include: - All biological children born to HCV-positive mothers. - Testing should consist of anti-HCV or HCV RNA testing. Because anti-HCV testing by itself prior to age 18 months may be falsely positive due to detection of passively acquired maternal antibody, testing prior to age 18 months should consist of HCV RNA testing. - Children with the same risk factors as adults. - Injection and intranasal drug use (current or former). - HIV-positive. - Signs or symptoms of liver disease (e.g., abnormal liver enzyme tests, jaundice, abdominal pain, swelling, fatigue). - Household contacts with HCV. - History of female genital cutting or mutilation (data are limited). - Screening tests that can be used are **antibody to HCV** (*anti-HCV*), **recombinant immunoblot assay** (*RIBA*), or **HCV RNA** polymerase chain reaction. Immunocompromised persons, such as those infected with HIV, those who have end-stage renal disease, and those receiving immunosuppressive therapy, may have false-negative tests and should be screened by HCV RNA testing.   For additional risk factors, screening considerations, visit the CDC website for updated information on HCV infection:  <https://www.cdc.gov/hepatitis/HCV/Management.htm> |
| **HIV** (93) | - Beginning January 4, 2010, refugees are no longer tested for HIV infection before arrival in the U.S. - **All refugee children > 12 years old should be screened unless they opt out.** - Screening should be repeated 3-6 months following resettlement for refugees who had recent exposure or are at high risk. - **Screen children < 13 years old** unless negative HIV status and the child is otherwise thought to be at low risk of infection (i.e., no history of high-risk exposures such as previous blood product transfusions, early sexual activity, or history of sexual violence or abuse). In most situations, complete risk information will not be available, and thus most children < 13 years of age should be screened. - **Screen children < 18 months of age** who test positive for HIV antibodies should receive further testing with DNA or RNA assays. Results of positive antibody tests in this age group can be unreliable because they may detect persistent maternal antibody (up to 18 months of age). - **All children born to or breast-fed by an HIV-infected mother should receive chemoprophylactic trimethoprim/sulfamethoxazole** beginning at 6 weeks of age until they are confirmed to be uninfected in order to prevent opportunistic infections. - Specific pediatric screening recommendations: - The American Academy of Pediatrics (AAP) recommends that infants born to HIV-positive mothers undergo DNA or RNA testing at day 14, again at 1-2 months of age, and then at 3-6 months of age.^94^ - A positive RNA or DNA result at any age is a presumptive indicator of HIV infection but must be confirmed. - The diagnosis of a HIV-infection is made if two DNA or RNA tests are positive. - CDC guidelines state that HIV is definitively excluded by two negative RNA or DNA tests (at 1 month and >4 months) or two negative antibody tests from separate specimens obtained at age >6 months. - Given that HIV can be transmitted from mother to child through breastfeeding, many clinicians confirm the absence of HIV-1 with a negative HIV-1 antibody assay at 12-18 months of age or after the child is no longer breastfeeding.   For additional information on HIV, visit the CDC website:  <https://www.cdc.gov/immigrantrefugeehealth/guidelines/domestic/screening-hiv-infection-domestic.html#ref-35> |
| **Malaria** (94) | - The most sensitive test for persons with subclinical malaria is polymerase chain reaction (PCR); when PCR is not available, traditional blood films or a rapid antigen test may be used but have limited sensitivity in asymptomatic persons. - All sub-Saharan Africa (SSA) refugees who arrived from countries that are endemic for *Plasmodium falciparum* should be assumed to have received pre-departure presumptive antimalarial therapy with artemisinin-based combination therapy unless they had a contraindication to treatment. Contraindications include pregnant individuals in their first trimester or lactating individuals, or infants weighing < 5 kg at the time of departure, individuals with a known allergy to the medication being used. - Refugees who **require** post-arrival testing or presumptive treatment, include: - SSA refugees receiving no presumptive treatment before departure (including those for whom presumptive treatment was contraindicated). - Any refugee from a malaria-endemic countries (SSA, Southeast Asia, eastern Mediterranean region, Mexico, Central and South America, South Pacific) with signs or symptoms of infection (should be referred immediately for specialty consultation). - Refugees who **do not require** post-arrival testing or presumptive treatment include: - SSA refugees receiving presumptive treatment before departure. - All refugees from malaria-endemic countries outside SSA.   For additional information on malaria, visit the CDC website:  <https://www.cdc.gov/immigrantrefugeehealth/guidelines/domestic/malaria-guidelines-domestic.html> |
| **Gonorrhea and**  **Chlamydia** (95) | Urine nucleic amplification test for the following:   - Females who are sexually active - Leukocyte esterase positive urine sample - Children with history of or at risk for sexual assault - Any refugee with symptoms |
| **Syphilis** (95) | Screening syphilis testing such as Venereal Disease Research Laboratory (VDRL ) or rapid plasma reagin (RPR) testing should be performed routinely for refugees in the following categories:   - All refugees > 15 years of age and older, if no overseas results are available. - Children < 15 years of age who are at risk for congenital syphilis (i.e., mother who tests positive for syphilis, if the mother’s syphilis results are not available, or the child is unaccompanied), who disclose sexual activity, or have been sexually assaulted should be evaluated. - Conduct confirmation testing for positive treponemal test results.   For additional information on disease specific sexually transmitted infections, visit the CDC website:  <https://www.cdc.gov/immigrantrefugeehealth/guidelines/domestic/sexually-transmitted-diseases/index.html> |
| **Intestinal and tissue invasive parasites (ITIP)** (28) | - Post-arrival screening for ITIP depends on the region of departure and predeparture presumptive therapy received. - Refugee children (> 1 year of age) from the Asia, Middle East, North Africa, Latin America, and Caribbean without contraindications receive a single dose of *albendazole* to treat soil-transmitted Helminths before departure. In addition to albendazole, sub-Saharan African (SSA) refugee children (> 4 year of age) without contraindications receive treatment with *praziquantel* for schistosomiasis. Refugee children currently receiving *presumptive therapy* for Strongyloides include those who do not live in Loa loa-endemic countries, children > 15 kg will receive *ivermectin* if they do not have contraindications. - For those who have **contraindications** (28) or **who did not receive complete pre-departure therapy** (28), the following ITIP screening is recommended at post-arrival initial domestic medical examination: - For refugees who had **no pre-departure presumptive treatment** or **incomplete presumptive treatment** - *Giardia lamblia* (all refugees): Conduct stool ova and parasites examination (two or more samples) if symptomatic (diarrhea, anorexia, poor weight gain/failure to thrive/malnutrition, abdominal distension); or provide presumptive treatment. - Roundworms/nematodes (all refugees): Conduct stool ova and parasites examination (two or more samples) or provide presumptive treatment. - Strongyloides (all refugees): Provide presumptive therapy or conduct diagnostics for Strongyloides (e.g., serologies for Strongyloides, two or more stool ova and parasites examinations, or Strongyloides culture method). - Schistosomiasis (SSA refugees): Provide presumptive therapy or conduct serologies for schistosomiasis (for SSA refugees who did not receive praziquantel). - CBC with differential (usually include absolute eosinophil count – caveat is that eosinophil is not sensitive or specific for invasive helminth parasitic infections, but if you have persistently elevated eosinophil count, will need further investigation). - For refugees who received **incomplete** presumptive treatment (28): - Strongyloides (all refugees): Provide presumptive therapy or conduct diagnostics for Strongyloides (e.g., serologies for Strongyloides, two or more stool ova and parasites examinations, or Strongyloides culture/ agar method). - Schistosomiasis: Provide presumptive therapy or conduct serologies for schistosomiasis (SSA refugees who did not receive praziquantel). - CBC with differential (usually include absolute eosinophil count – caveat is that eosinophil is not sensitive or specific for invasive helminth parasitic infections, but if you have persistently elevated eosinophil count, will need further investigation). - For refugees who received **complete predeparture presumptive treatment:** - Absolute eosinophil count (routinely recommended as part of the hematology testing and is not sensitive or specific for invasive parasites, but a persistently elevated count indicates the need for further investigation)   For more details on intestinal parasites, resources, and summary recommendations for post-arrival management, visit the CDC: <https://www.cdc.gov/immigrantrefugeehealth/guidelines/domestic/intestinal-parasites-domestic.html> |
| **Tuberculosis** (17) | - Pre-departure screening results and treatment information should accompany the refugee to the initial domestic medical examination (in the International Organization for Migration bag). Overseas TB screening results, treatment and classifications are documented on official U.S. Department of State form. Also provided through the Electronic Disease Notification (EDN) system to individual state health departments. - Tuberculosis is one of the most common infectious diseases in refugees and all refugee applicants should be screened after arrival. - Any refugee, regardless of country of origin, with signs or symptoms of TB disease should undergo clinical evaluation for TB disease. - Domestic TB evaluation for newly arrived refugee children from endemic countries (17) - **For children aged < 2 years:** - A tuberculin skin test (TST) is recommended (if not previously treated for LTBI or TB disease). - **For children aged 2-14 years:** - If interferon gamma release assay (IGRA) was negative overseas (within the last 6 months), and there are no signs or symptoms of TB disease, no further domestic evaluation is needed. - If the overseas IGRA was negative but performed > 6 months prior to the domestic medical examination, repeat IGRA. - Treatment for latent tuberculosis infection (LTBI) should be considered if TB disease is ruled out for those with positive IGRA results unless TB disease or LTBI treatment was completed prior to arrival. - **For children aged > 15 years**: - If the overseas IGRA was not done overseas or a negative IGRA was documented > 6 months prior, an IGRA is recommended at the domestic examination. - If overseas or domestic IGRA is positive, LTBI treatment should be considered after TB disease is ruled out (if not previously treated for LTBI or TB disease).   Note: TST and any vaccine can be administered on the same day.  If the patient received a live injectable vaccine (e.g., measles-mumps-rubella or varicella) the previous day or earlier, TST must be delayed for at least 4 weeks (no need to wait if the TST was placed first).  For more details on tuberculosis, classifications, travel clearance, and domestic refugee screening for TB, visit the CDC: <https://www.cdc.gov/immigrantrefugeehealth/guidelines/domestic/tuberculosis-guidelines.html> |

References

1. United Nations High Commissioner for Refugees (UNHCR). UNHCR mid-year trends. (2022). Available at: https://www.unhcr.org/statistics/unhcrstats/635a578f4/mid- year-trends-2022.html (accessed June 3, 2023).

2. UNICEF. Child displacement-UNICEF. Data: June 2022. Available at: https://www. unicef.org/eap/press-releases/nearly-37-million-children-displaced-worldwide-highest- number-ever-recorded#:~:text=NEW%20YORK%2C%2017%20June%202022,since%20 the%20Second%20World%20War (accessed June 3, 2023).

3. Page MJ, McKenzie JE, Bossuyt PM, Boutron I, Hoffmann TC, Mulrow CD, et al. The PRISMA 2020 statement: an updated guideline for reporting systematic reviews. *BMJ*. (2021) 372:n71. doi: 10.1136/bmj.n71

4. Dawson-Hahn EE, Pak-Gorstein S, Hoopes AJ, Matheson J. Comparison of the nutritional status of overseas refugee children with low-income children in Washington state. *PLoS One*. (2016) 11:e0147854. doi: 10.1371/journal.pone.0147854

5. WHO Multicenter Growth Reference Study Group. WHO child growth standards based on length/height, weight and age. *Acta Pediatric Suppl*. (2006) 95:76–85. doi: 10.1111/j.1651-2227.2006.tb02378.x

6. Centers for Disease Control and Prevention (CDC). Guidance for evaluating nutritional status and growth in refugee children during the domestic medical screening examination. In: Centers for Disease Control and Prevention, immigrant, refugee, and migrant health. Available at: https://www.cdc.gov/immigrantrefugeehealth/guidelines/ domestic/nutrition-growth.html (accessed April 15, 2022).

7. Hervey K, Vargas D, Klesges L, Fischer PR, Trippel S, Juhn YJ. Overweight among refugee children after arrival in the United States. *J Health Care Poor Underserved*. (2009) 20:246–56. doi: 10.1353/hpu.0.0118

8. Pernitez-Agan S, Wickramage K, Yen C, Dawson-Hahn E, Mitchell T, Zenner D. Nutritional profile of Syrian refugee children before resettlement. *Confl Heal*. (2019) 13:22. doi: 10.1186/s13031-019-0208-y

9. Goel MS, McCarthy EP, Phillips RS, Wee CC. Obesity among US immigrant subgroups by duration of residence. *JAMA*. (2004) 292:2860–7. doi: 10.1001/ jama.292.23.2860

10. World Health Organization (WHO). *Emergencies preparedness, nutrition and food safety. The management of nutrition in major emergencies*. Geneva: World Health Organization (2000).

11. Ziersch A, Walsh M, Due C, Duivesteyn E. Exploring the relationship between housing and health for refugees and asylum seekers in South Australia: a qualitative study. *Int J Environ Res Public Health*. (2017) 14:1036. doi: 10.3390/ijerph14091036

12. Grammatikopoulou MG, Theodoridis X, Poulimeneas D, Maraki MI, Gkiouras K, Tirodimos I, et al. Malnutrition surveillance among refugee children living in reception centers in Greece: a pilot study. *Int Health*. (2019) 11:30–5. doi: 10.1093/inthealth/ihy053

13. Baauw A, Kist-van Holthe J, Slattery B, Heymans M, Chinapaw M, van Goudoever H. Health needs of refugee children identified on arrival in reception countries: a systematic review and meta-analysis. *BMJ Paediatr Open*. (2019) 3:e000516. doi: 10.1136/bmjpo-2019-000516

14. Centers for Disease Control and Prevention (CDC). Vitamin B12 deficiency in resettled Bhutanese refugees – United States, 2008-2011. *MMWR Morb Mortal Wkly Rep* (2011);60:343–346. Available at: https://www.cdc.gov/mmwr/preview/mmwrhtml/ mm6011a4.htm (accessed April 17, 2022).

15. Schiergens KA, Staudigl M, Borggraefe I, Maier EM. Neurological sequelae due to inborn metabolic diseases in pediatric refugees: challenges in treating the untreated. *Neuropediatrics*. (2018) 49:363–8. doi: 10.1055/s-0038-1661415

16. Annamalai A. (2014). *Refugee health care: An essential medical guide*. Springer Science & Business, New York. *2nd*, 29–41.

17. Centers for Disease Control and Prevention (CDC). Guidance for screening for tuberculosis infection and disease during domestic medical examination for newly arrived refugees. In: Centers for Disease Control and Prevention, immigrant, refugee, and migrant health. Available at: https://www.cdc.gov/immigrantrefugeehealth/ guidelines/domestic/tuberculosis-guidelines.html#ref-1 (accessed April 16, 2022).

18. Collins JM, Stout JE, Ayers T, Hill AN, Katz DJ, Ho CS, et al. Winglee K; tuberculosis epidemiologic studies consortium. Prevalence of latent tuberculosis infection among non-US-born persons by country of birth-United States, 2012-2017. *Clin Infect Dis*. (2021) 73:e3468–75. doi: 10.1093/cid/ciaa1662

19. Taylor EM, Painter J, Posey DL, Zhou W, Shetty S. Latent tuberculosis infection among immigrant and refugee children arriving in the United States: 2010. *J Immigr Minor Health*. (2016) 18:966–70. doi: 10.1007/s10903-015-0273-2

20. Pai M, Denkinger CM, Kik SV, Rangaka MX, Zwerling A, Oxlade O, et al. Gamma interferon release assays for detection of *Mycobacterium tuberculosis* infection. *Clin Microbiol Rev*. (2014) 27:3–20. doi: 10.1128/CMR.00034-13

21. Metcalfe JZ, Cattamanchi A, McCulloch CE, Lew JD, Ha NP, Graviss EA. Test variability of the QuantiFERON-TB gold in-tube assay in clinical practice. *Am J Respir Crit Care Med*. (2013) 187:206–11. doi: 10.1164/rccm.201203-0430OC

22. Yun K, Urban K, Mamo B, Matheson J, Payton C, Scott KC, et al. Increasing hepatitis B vaccine prevalence among refugee children arriving in the United States, 2006-2012. *Am J Public Health*. (2016) 106:1460–2. doi: 10.2105/AJPH.2016.303203

23. Mitruka K, Pezzi C, Baack B, Burke H, Cochran J, Matheson J, et al. Evaluation of hepatitis B virus screening, vaccination, and linkage to care among newly arrived refugees in four states, 2009-2011. *J Immigr Minor Health*. (2019) 21:39–46. doi: 10.1007/ s10903-018-0705-x

24. Centers for Disease Control and Prevention (CDC). Screening for viral hepatitis during domestic medical examination of newly arrived refugees. In: Centers for Disease Control and Prevention, immigrant, refugee, and migrant health. Available at: https:// www.cdc.gov/immigrantrefugeehealth/guidelines/domestic/hepatitis-screening- guidelines.html (accessed April 16, 2022).

25. Mody R. Chapter 20: intestinal parasites In: PF Walker and ED Barnett, editors. *Immigrant Medicine*. Edinburg, Scotland: W.B. Saunders (2007). 273–307.

26. Webster J, Stauffer W, Mitchell T, Lee D, O'Connell E, Weinberg M, et al. Cross-sectional assessment of the Association of Eosinophilia with intestinal parasitic infection in U.S.-bound refugees in Thailand: prevalent, age dependent, but of limited clinical
utility. *Am J Trop Med Hyg*. (2022) 106:1552–9. doi: 10.4269/ajtmh.21-0853

27. Swanson SJ, Phares CR, Mamo B, Smith KE, Cetron MS, Stauffer WM. Albendazole therapy and enteric parasites in United States-bound refugees. *N Engl J Med*. (2012) 366:1498–507. doi: 10.1056/NEJMoa1103360

28. Centers for Disease Control and Prevention (CDC). Overseas refugee health guidance. In: Centers for Disease Control and Prevention, immigrant, refugee, and migrant health. Intestinal parasite guidance: Strongyloidiasis, schistosomiasis, and soil-transmitted helminth infections. Available at: https://www.cdc.gov/ immigrantrefugeehealth/guidelines/overseas-guidelines.html#ipg (accessed March 6, 2023).

29. Parker DM, Carrara VI, Pukrittayakamee S, McGready R, Nosten FH. Malaria ecology along the Thailand-Myanmar border. *Malar J*. (2015) 14:388. doi: 10.1186/ s12936-015-0921-y

30. Mitchell T, Dalal W, Klosovsky A, Yen C, Phares C, Burkhardt M, et al. An immunization program for US-bound refugees: development, challenges, and opportunities 2012-present. *Vaccine*. (2021) 39:68–77. doi: 10.1016/j.vaccine.2020.10.047

31. Lam E, McCarthy A, Brennan M. Vaccine-preventable diseases in humanitarian emergencies among refugee and internally displaced populations. *Hum Vaccin* 1004 *Immunother*. (2015) 11:2627–36. doi: 10.1080/21645515.2015.1096457

32. Kouadio IK, Koffi AK, Attoh-Toure H, Kamigaki T, Oshitani H. Outbreak of measles and rubella in refugee transit camps. *Epidemiol Infect*. (2009) 137:1593–601. doi: 10.1017/S0950268809002520

33.World Health Organization (WHO). Regional Office for the Eastern Mediterranean: Afghanistan. Measles vaccination campaign kicks off in Afghanistan to fight ongoing outbreak: Around 1.2 million children in 24 provinces to get vaccinated.
Available at: http://www.emro.who.int/afg/afghanistan-news/measles-vaccination-kicks-off-in-afghanistan-to-fight-ongoing-outbreak-around-12-million-children-in-24-provinces-to-get-vaccinated.html (accessed April 19, 2022).

34. Jakab Z. (2020). World Health Organization: Vaccines work at all ages, everywhere. Available at: https://www.who.int/news-room/commentaries/detail/vaccines-work-at-all-ages-everywhere/ (accessed February 26, 2023).

35. Centers for Disease Control and Prevention (CDC). Screening for Lead during domestic medical examination for newly arrived refugees. In: Centers for Disease Control and Prevention, immigrant, refugee, and migrant health. Available at: https://www.cdc.gov/immigrantrefugeehealth/guidelines/lead-guidelines.html (accessed April 17, 2022).

36. Pezzi C, Lee D, Kennedy L, Aguirre J, Titus M, Ford R, et al. Blood Lead levels among resettled refugee children in select US states, 2010-2014. *Pediatrics*. (2019) 143:e20182591. doi: 10.1542/peds.2018-2591

37. Seifu S, Tanabe K, Hauck FR. The prevalence of elevated blood Lead levels in foreign-born refugee children upon arrival to the U.S. and the adequacy of follow-up treatment. *J Immigr Minor Health*. (2020) 22:10–6. doi: 10.1007/s10903-019-00878-6

38. Yun K, Matheson J, Payton C, Scott KC, Stone BL, Song L, et al. Health profiles of newly arrived refugee children in the United States, 2006-2012. *Am J Public Health*. (2016) 106:128–35. doi: 10.2105/AJPH.2015.302873

39. Mellin-Sanchez L, Sondheimer N. An Infant Refugee with Anemia and Low Serum Vitamin B12. *Clin. Chem.* (2018) 64:1567–70. doi: 10.1373/clinchem.2017.283283

40. The Lancet Hematology. Editorial: meeting the health needs of people from Afghanistan. *Lancet*. (2022) 9:E167. doi: 10.1016/S2352-3026(22)00051-5

41. Kay A, Leidman E, Lopez V, Wilkinson C, Tondeur M, Bilukha O. The burden of anemia among displaced women and children in refugee settings worldwide, 2013-2016. *BMJ Glob Health*. (2019) 4:e001837. doi: 10.1136/bmjgh-2019-001837

42. Crespo E. The importance of Oral health in immigrant and refugee children. *Children (Basel)*. (2019) 6:102. doi: 10.3390/children6090102

43. Paisi M, Baines R, Burns L, Plessas A, Radford P, Shawe J, et al. Barriers and facilitators 1032 to dental care access among asylum seekers and refugees in highly developed countries: a systematic review. *BMC Oral Health*. (2020) 20:337. doi: 10.1186/s12903-020-01321-1

44. United Nations. Ukraine war linked to massive malnutrition crisis affecting millions in other emergencies. Available at: https://news.un.org/en/story/2022/04/1115762 (accessed April 15, 2022).

45. Lutfy C, Cookson ST, Talley L, Rochat R. Malnourished children in refugee camps and lack of connection with services after US resettlement. *J Immigr Minor Health*. (2014) 16:1016–22. doi: 10.1007/s10903-013-9796-6

46. Dawson-Hahn E, Pak-Gorstein S, Matheson J, Zhou C, Yun K, Scott K, et al. Growth trajectories of refugee and nonrefugee children in the United States. *Pediatrics*. (2016) 138:e20160953. doi: 10.1542/peds.2016-0953

47. Heney JH, Dimock CC, Friedman JF, Lewis C. Pediatric refugee in Rhode Island increases in BMI percentile, overweight, and obesity following resettlement. *R I Med J*. (2013) 98:43–7.

48. Olson BG, Kurland Y, Rosenbaum PF, Hobart TR. Rapid weight gain in pediatric refugees after US immigration. *J Immigrant Minority Health*. (2017) 19:263–6. doi: 10.1007/s10903-016-0461-8

49. Kelsey MM, Zaepfel A, Bjornstad P, Nadeau KJ. Age-related consequences of childhood obesity. *Gerontology*. (2014) 60:222–8. doi: 10.1159/000356023

50. Global Food Security Cluster. (2021). GFSC statement on averting famine: We have reached a critical time for action. Available at: https://fscluster.org/news/global- food-security-cluster-statement (accessed March 5, 2023).

51. Nisbet C, Lestrat KE, Vatanparast H. Food security interventions among refugees around the globe: a scoping review. *Nutrients*. (2022) 14:522. doi: 10.3390/nu14030522

52. United Nations World Food Programme. (2023). Help the world food programme save lives around the world. Available at: https://secure.wfpusa.org/donate/Brand- Search_SRCH?ms=Brand-Search_SRCH_GSA_Brand-IS_Brand_EvergreenSearch_ AD&gclid=EAIaIQobChMIyfLP67nI_QIVhRPUAR36XAb5EAAYASAAEgL1Y_D_ BwE (accessed March 5, 2023).

53. Kaur S, Bhatia S, Ganguly N. Tuberculosis diagnosis in the era of molecular diagnostics: challenges and progress. *Indian J Med Res*. (2019) 150:384–94.

54. Centers for Disease Control and Prevention (CDC). (2016). Reported tuberculosis in the United States: Tuberculosis, data and statistics. Available at: https://www.cdc.gov/ tb/statistics/reports/2016/default.htm (accessed February 20, 2023).

55. Khan A, Phares CR, Phuong HL, Trinh DTK, Phan H, Merrifield C, et al. Overseas treatment of latent tuberculosis infection in US-bound immigrants. *Emerg Infect Dis*. (2022) 28:582–90. doi: 10.3201/eid2803.212131

56. Cain KP, Marano N, Kamene M, Sitienei J, Mukherjee S, Galev A, et al. The movement of multidrug-resistant tuberculosis across borders in East Africa needs a regional and global solution. *PLoS Med*. (2015) 12:e1001791. doi: 10.1371/journal.pmed.1001791

57. Ismail MB, Rafei R, Dabboussi F, Hamze M. Tuberculosis, war, and refugees: spotlight on the Syrian humanitarian crisis. *PLoS Pathog*. (2018) 14:e1007014. doi: 10.1371/journal.ppat.1007014

58. Priebe S, Giacco D, El-Nagib R. *Public health aspects of mental health among migrants and refugees: A review of the evidence on mental health care for refugees, asylum seekers, and irregular migrants in the WHO European region*. Copenhagen: WHO Regional Office for Europe (2016).

59. Herrera-Molina M, González-Sánchez DF, Díaz-Sánchez D, Fernández-de-Castro J. Tuberculosis in Guatemala: An epidemiological update. *Tropical Med Infect Dis*. (2020) 5:116.

60. Kumar M, Mishra A, Mohanty S, Prakash A, Mishra A. Tuberculosis status in India: an overview. *Indian J Tuberc*. (2020) 67:79–90.

61. Madzingira E, Zvandasara M, Chikobvu P, Chagwedera T. Tuberculosis burden and response in Zimbabwe: a review of the current status and future prospects. *Pan Afr Med J*. (2020) 36:71.

62. McColl SP, Akbar K, Hussain SR, Singh S. Tuberculosis in Afghanistan: epidemiology, treatment, and challenges. *Lancet Glob Health*. (2019) 7:e1178–88.

63. Moges BY, Gebremariam M, Erko B. Tuberculosis in Ethiopia: epidemiology, prevention, and control. *Int J Environ Res Public Health*. (2019) 16:3896.

64. Mondul A, Thon K, Jokam A, Panyako B, Monyo E, Makur A. Tuberculosis burden and progress towards achieving global tuberculosis elimination goals in South Sudan: a review. *PLoS One*. (2020) 15:e0235978

65. World Health Organization. (2020). Tuberculosis. Available at: https://www.who. int/news-room/fact-sheets/detail/tuberculosis (accessed February 22, 2023).

66. Mitchell T, Weinberg M, Posey DL, Cetron M. Immigrant and refugee health: a Centers for Disease Control and Prevention perspective on protecting the health and health security of individuals and communities during planned migrations. *Pediatr Clin* 1096 *N Am*. (2019) 66:549–60. doi: 10.1016/j.pcl.2019.02.004

67. Schwartzman K, Oxlade O, Barr G, Grimard F, Acosta I, Baez J, et al. Domestic returns from investment in the control of tuberculosis in other countries. *N Engl J Med*.(2005) 353:1008–20. doi: 10.1056/NEJMsa043194 1099

68. Douglas P, Posey DL, Zenner D, Robson J, Abubakar I, Giovinazzo G. Capacity 1100 strengthening through premigration tuberculosis screening programmes: IRHWG experiences. *Int J Tuberc Lung Dis*. (2017) 21:737–45. doi: 10.5588/ijtld.17.0019

69. Dara M, de Colombani P, Petrova-Benedict R, Centis R, Zellweger JP, Sandgren A, et al. Wolfheze Transborder migration task force. Minimum package for cross-border 1103 TB control and care in the WHO European region: a Wolfheze consensus statement. *Eur Respir J*. (2012) 40:1081–90. doi: 10.1183/09031936.00053012

70. ERS-WHO e-Consilium. Copenhagen: WHO regional office for Europe. (2018).
Available at: https://www.euro.who.int/en/health-topics/communicable-diseases/tuberculosis/areas-of-work/technical-cooperation/ers-who-e-consilium (accessed April 14, 2022).

71. Dara M, Sulis G, Centis R, D'Ambrosio L, de Vries G, Douglas P, et al. Cross-border collaboration for improved tuberculosis prevention and care: policies, tools and 1109 experiences. *Int J Tuberc Lung Dis*. (2017) 21:727–36. doi: 10.5588/ijtld.16.0940

72. Mukhtar NA, Kathpalia P, Hilton JF, Lau G, Yu A, Grumbach K, et al. Provider, patient, and practice factors shape hepatitis B prevention and management by primary care providers. *J Clin Gastroenterol*. (2016) 51:626–31. doi: 10.1097/MCG.0000000000000738

73. Mishori R, Aleinikoff S, Davis D. Primary Care for Refugees: challenges and opportunities. *Am Fam Physician*. (2017) 96:112–20.

74. Ortega-Sanchez IR, Vijayaraghavan M, Barskey AE, Wallace GS. The economic burden of sixteen measles outbreaks on United States public health departments in 2011. *Vaccine*. (2014) 32:1311–7. doi: 10.1016/j.vaccine.2013.10.012

75. Office of Refugee Resettlement. Revised medical screening guidelines for newly arriving refugees. (2013). Available at: https://www.acf.hhs.gov/orr/policy-guidance/revised-medical-screening-guidelines-newly-arriving-refugees (accessed April 18, 2022).

76. Andersen M, Kruse A, Frederiksen H, Ahmadi A, Norredam M. Health status of newly resettled in Denmark. *Dan Med J*. (2020) 67:A08200567.

77. Centers for Disease Control and Prevention (CDC). Immigrant, refugee, and migrant health. Refugee health profiles. Available at: https://www.cdc.gov/immigrantrefugeehealth/profiles/index.html (accessed September 2, 2023).

78. Frederiksen NW, Christoffersen NM, Haugaard AK, Ahmadi A, Poulsen A, Norredam M, et al. Health screening among children newly granted asylum in Denmark. *Acta Paediatr*. (2021) 110:2389–95. doi: 10.1111/apa.15879

79. Oberg C. The arc of migration and the impact on Children's health and well-being
forward to the special issue-children on the move. *Children (Basel)*. (2019) 6:100. doi: 10.3390/children6090100

80. European Website on Integration. Denmark: The integration act (as amended). October 2017. Available at: https://ec.europa.eu/migrant-integration/library-document/ integration-act-amended-october-2017_en (accessed April 17, 2022).

81. Fazel M, Reed RV, Panter-Brick C, Stein A. Mental health of displaced and refugee children resettled in high-income countries: risk and protective actors. *Lancet*. (2012) 379:266–82. doi: 10.1016/S0140-6736(11)60051-2

82. Maldari T, Elsley N, Abdul RR. The health status of newly arrived Syrian refugees at the refugee health service, South Australia, 2016. *Aust J Gen Pract*. (2019) 48:480–6. doi: 10.31128/AJGP-09-18-4696

83. Montgomery E. Trauma, exile and mental health in young refugees. *Acta Psychiatr* *Scand Suppl*. (2011) 124:1–46. doi: 10.1111/j.1600-0447.2011.01740.x

84. Yale Humanitarian Research Lab. Yale University School of Public Health. (2023). Russia’s systematic program for the re-education and adoption of Ukraine’s children. Available at: https://hub.conflictobservatory.org/portal/apps/sites/#/home/pages/children-camps-1 (accessed February 27, 2023).

85. Phares CR, Liu Y, Wang Z, et al. Disease Surveillance Among U.S.-Bound Immigrants and Refugees — Electronic Disease Notification System, United States, 2014–2019. *MMWR Surveill Summ.* (2022) 71(No. SS-2):1–21. doi: <http://dx.doi.org/10.15585/mmwr.ss7102a1>

86. Centers for Disease Control and Prevention (CDC). Guidelines for the US domestic medical examination for newly arriving refugees Available at: <https://www.cdc.gov/immigrantrefugeehealth/guidelines/domestic-guidelines.html> (accessed February 20, 2023).

87. Centers for Disease Control and Prevention (CDC). General and optional testing for newly arrived refugees. In: Centers for Disease Control and Prevention, Immigrant, Refugee, and Migrant Health. Available at: <https://www.cdc.gov/immigrantrefugeehealth/guidelines/domestic/general/index.html> (accessed March 6, 2023).

88. Chilton LA, Gilbert HA, Paz-Soldan GJ, Granado-Villar DC, Gitterman BA, et al. Providing Care for Immigrant, Migrant, and Border Children. Pediatrics. (2013) 131(6):e2028–e2034. doi: 10.1542/peds.2013-1099

89. Centers for Disease Control and Prevention. Recommendations to Prevent and Control Iron Deficiency in the United States. MMWR 1998;47(No. RR-3). Available at: <https://www.cdc.gov/mmwr/pdf/rr/rr4703.pdf> (accessed March 5, 2023).

90. World Health Organization (WHO). Guideline: vitamin A supplementation in infants and children 6-59 months of age; 2011. Available at: <https://www.who.int/publications/i/item/9789241501767> (accessed March 5, 2023).

91. Caulfield L, Black R. Zinc deficiency. In: Ezzati M, Lopez A, Rodgers A, Murray C, eds. Comparative Quantification of Health Risks: Global and Regional Burden of Disease Attributable to Selected Major Risk Factors. Geneva, Switzerland: WHO; 2004.

92. Weinbaum CM, Mast EE, Ward JW. Recommendations for identification and public health management of persons with chronic hepatitis B virus infection. *Hepatology*. (2009) 49(5 Suppl):S35-44. doi: 10.1002/hep.22882

93. Centers for Disease Control and Prevention. Screening for HIV infection during the refugee domestic medical examination. Available at: <https://www.cdc.gov/immigrantrefugeehealth/guidelines/domestic/screening-hiv-infection-domestic.html> (accessed March 6, 2023).

94. Centers for Disease Control and Prevention (CDC). Malaria. In: Centers for Disease Control and Prevention, immigrant, refugee, and migrant health. Available at https://www.cdc.gov/immigrantrefugeehealth/guidelines/domestic/malaria-guidelines-domestic.html (accessed March 6, 2023).

95. Centers for Disease Control and Prevention (CDC). Sexual and reproductive health screening during the domestic medical examination for newly arrived refugees. Available at: <https://www.cdc.gov/immigrantrefugeehealth/guidelines/domestic/sexually-transmitted-diseases/index.html> (accessed March 6, 2023).
